# Supplementary material for: Altered Brain Structure in an ATRX‐Deficient Mouse Model of Autism Spectrum Disorder
Source: Autism Res. 2026 Feb 22;19(4):e70205. doi: 10.1002/aur.70205 (PMC13087846; doi:10.1002/aur.70205)
Supplement: Supplementary file 1 — Figure S1: Graphical representation of relative volumes of hippocampal subregions in male and female control and AtrxNEXCre mice. Figure S2: Graphical representation of relative volumes of cortical subregions in male and female control and AtrxNEXCre mice. Figure S3: Graphical representation of relative volumes of cerebellar subregions in male and female control and AtrxNEXCre mice. Figure S4: Representative images of brain regions showing NEXCre expressing cells labeled with SUN1‐GFP and co‐labeled with ATRX, demonstrating regions with ATRX loss vs. ATRX retained expression. Data S1: Raw MRI data. Excel file containing raw absolute volumes and relative volume outputs, as well as statistical analysis across all brain regions. [file AUR-19-0-s001.zip › Supplemental Fig 1.pdf]

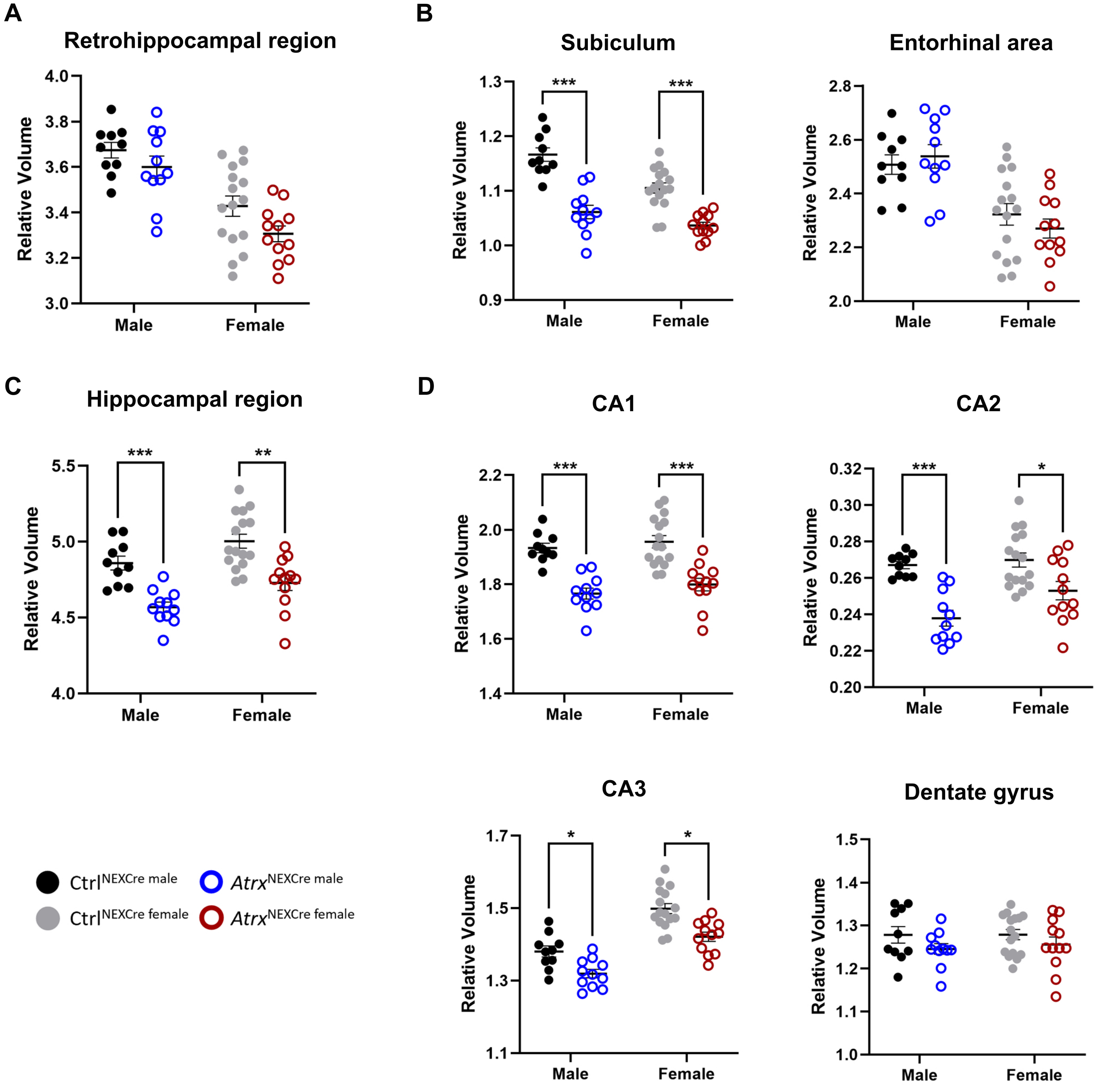


**Supplemental Figure 1: Relative volume changes in subregions of the hippocampal formation in Atrx$^{NexCre}$ mice.** **A)** No change in the retrohippocampal formation, but **B)** subregions of the retrohippocampal region showed a significant decrease in the subiculum but not the entorhinal area. **C)** The hippocampal region had a decrease in relative volume, along with the **D)** CA1, CA2, and CA3 but not the dentate gygrus. (* = FDR <0.10, ** = FDR <0.05, *** = FDR <0.001, **** = FDR <0.0001). (Ctrl$^{male}$ n=10, *Atrx*$^{NEXCre\ male}$ n=11, Ctrl$^{female}$ n=16, *Atrx*$^{NEXCre\ female}$ n=12).
