# Supplementary material for: Altered Brain Structure in an ATRX‐Deficient Mouse Model of Autism Spectrum Disorder
Source: Autism Res. 2026 Feb 22;19(4):e70205. doi: 10.1002/aur.70205 (PMC13087846; doi:10.1002/aur.70205)
Supplement: Supplementary file 1 — Figure S1: Graphical representation of relative volumes of hippocampal subregions in male and female control and AtrxNEXCre mice. Figure S2: Graphical representation of relative volumes of cortical subregions in male and female control and AtrxNEXCre mice. Figure S3: Graphical representation of relative volumes of cerebellar subregions in male and female control and AtrxNEXCre mice. Figure S4: Representative images of brain regions showing NEXCre expressing cells labeled with SUN1‐GFP and co‐labeled with ATRX, demonstrating regions with ATRX loss vs. ATRX retained expression. Data S1: Raw MRI data. Excel file containing raw absolute volumes and relative volume outputs, as well as statistical analysis across all brain regions. [file AUR-19-0-s001.zip › Supplemental Fig 2.pdf]

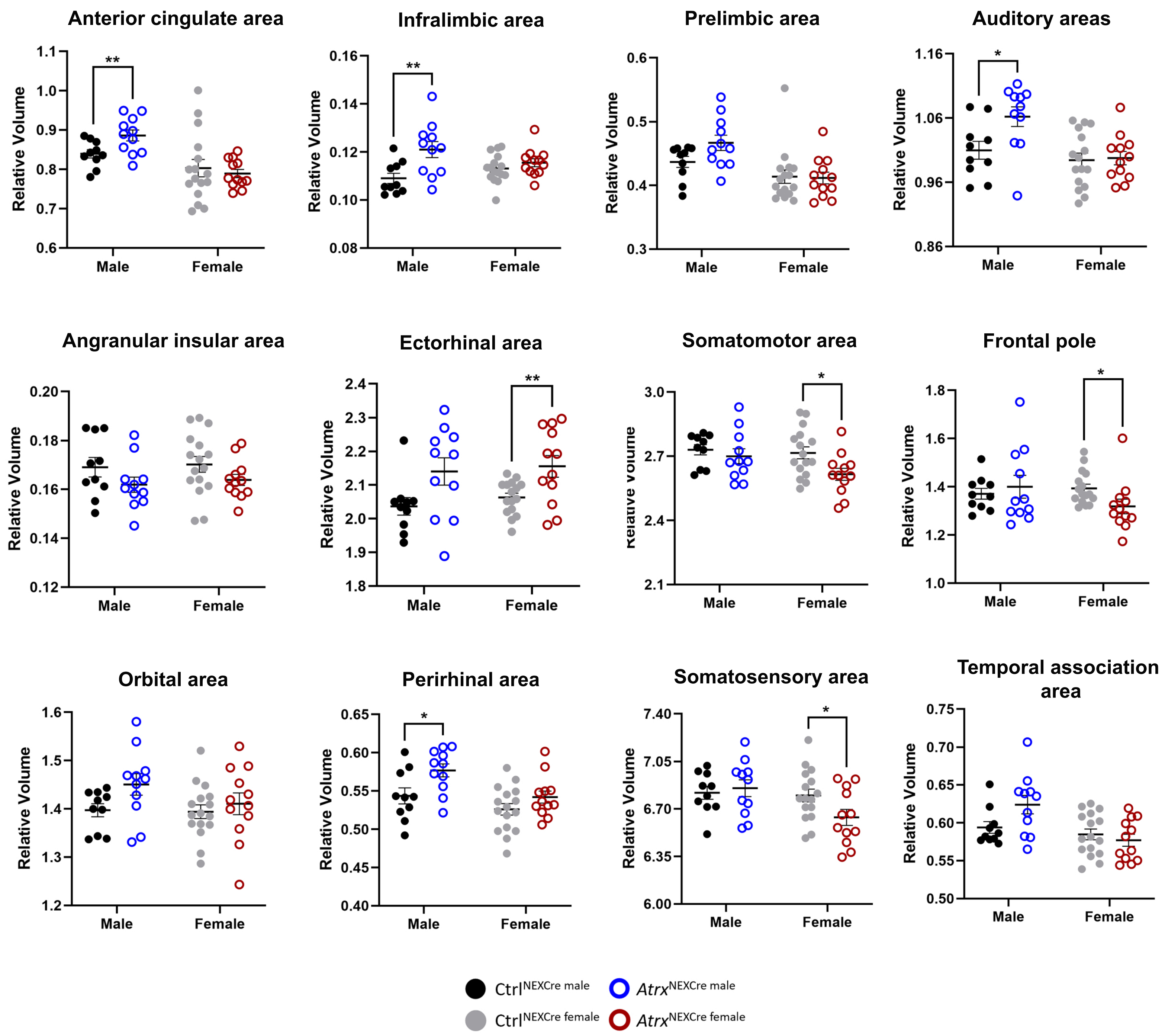

Supplemental Figure 2: Changes in relative volumes of cortical regions in $Atrx^{NexCre}$ mice. Male $Atrx^{NEXCre}$ mice have an increase in relative volumes in the anterior cingulate area, infralimbic area, auditory area, and perirhinal area. Female mice show an increase in relative volume in the ectorhinal area, and a decrease in relative volume in the somatomotor area, frontal pole, and somatosensory area. (* = FDR <0.10, ** = FDR <0.05) ($Ctrl^{male}$ n=10, $Atrx^{NEXCre\ male}$ n=11, $Ctrl^{female}$ n=16, $Atrx^{NEXCre\ female}$ n=12).
