# Supplementary material for: Altered Brain Structure in an ATRX‐Deficient Mouse Model of Autism Spectrum Disorder
Source: Autism Res. 2026 Feb 22;19(4):e70205. doi: 10.1002/aur.70205 (PMC13087846; doi:10.1002/aur.70205)
Supplement: Supplementary file 1 — Figure S1: Graphical representation of relative volumes of hippocampal subregions in male and female control and AtrxNEXCre mice. Figure S2: Graphical representation of relative volumes of cortical subregions in male and female control and AtrxNEXCre mice. Figure S3: Graphical representation of relative volumes of cerebellar subregions in male and female control and AtrxNEXCre mice. Figure S4: Representative images of brain regions showing NEXCre expressing cells labeled with SUN1‐GFP and co‐labeled with ATRX, demonstrating regions with ATRX loss vs. ATRX retained expression. Data S1: Raw MRI data. Excel file containing raw absolute volumes and relative volume outputs, as well as statistical analysis across all brain regions. [file AUR-19-0-s001.zip › Supplemental fig 3.pdf]

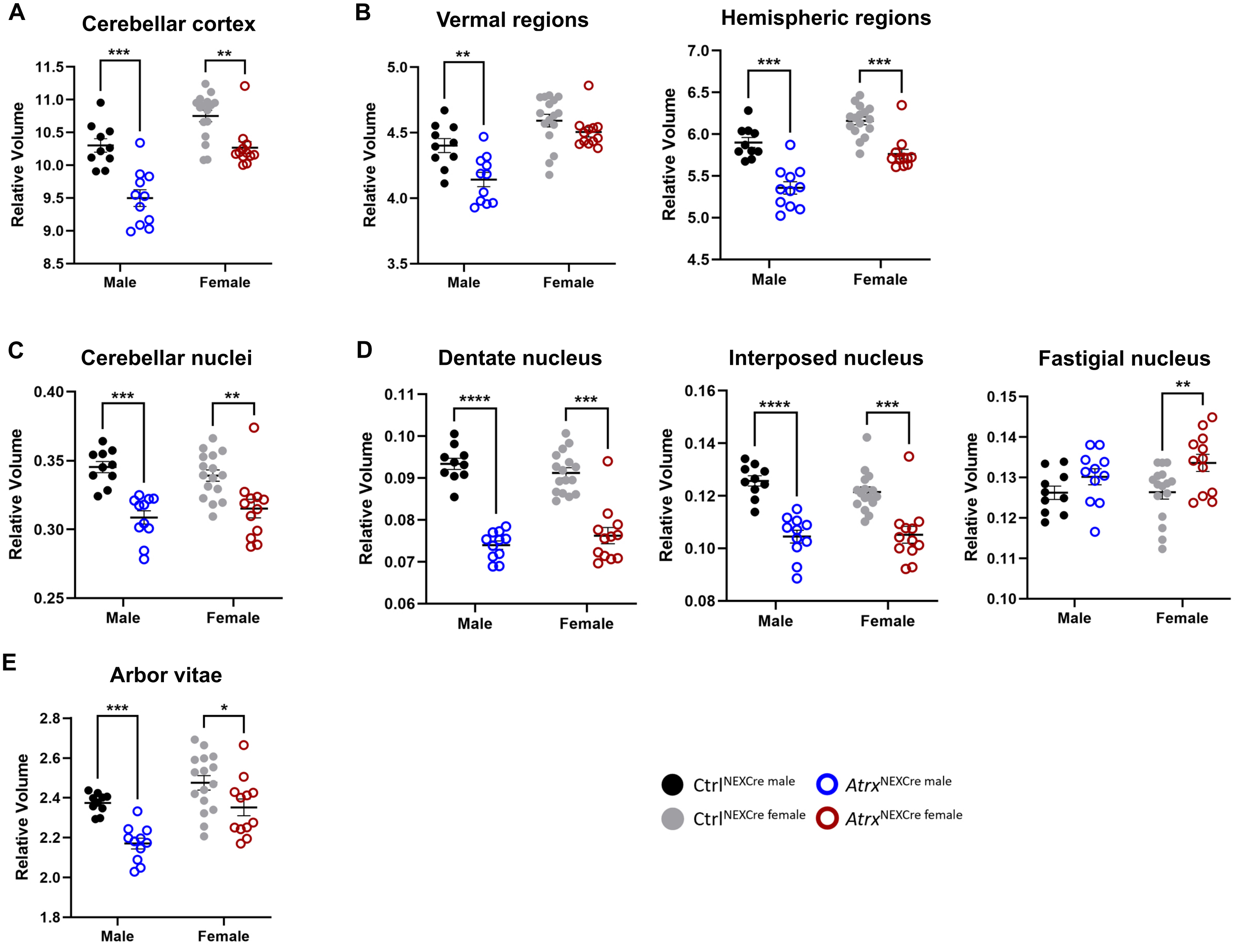

Supplemental Figure 3: Alterations in relative volumes of cerebellar subregions in Atrx^{NexCre} mice.
A) Decreased relative volume of the cerebellar cortex and **B)** the cerebellar cortex subregions vermal and hemispheric. **C)** A decrease in total cerebellar nuclei relative volume and **D)** the cerebellar nuclei, dentate nucleus and interposed nucleus, with an increased relative volume in the fastigial nucleus in females only. **E)** A decrease in relative volume of the cerebellar arbor vitae. (* = FDR <0.10, ** = FDR <0.05, *** = FDR <0.001,**** FDR = <0.0001) (Ctrl^{male} n=10, *Atrx*$^{NEXCre\ male}$ n=11, Ctrl^{female} n=16, *Atrx*$^{NEXCre\ female}$ n=12).
