# Supplementary material for: Altered Brain Structure in an ATRX‐Deficient Mouse Model of Autism Spectrum Disorder
Source: Autism Res. 2026 Feb 22;19(4):e70205. doi: 10.1002/aur.70205 (PMC13087846; doi:10.1002/aur.70205)
Supplement: Supplementary file 1 — Figure S1: Graphical representation of relative volumes of hippocampal subregions in male and female control and AtrxNEXCre mice. Figure S2: Graphical representation of relative volumes of cortical subregions in male and female control and AtrxNEXCre mice. Figure S3: Graphical representation of relative volumes of cerebellar subregions in male and female control and AtrxNEXCre mice. Figure S4: Representative images of brain regions showing NEXCre expressing cells labeled with SUN1‐GFP and co‐labeled with ATRX, demonstrating regions with ATRX loss vs. ATRX retained expression. Data S1: Raw MRI data. Excel file containing raw absolute volumes and relative volume outputs, as well as statistical analysis across all brain regions. [file AUR-19-0-s001.zip › Supplemental Fig 4.pdf]

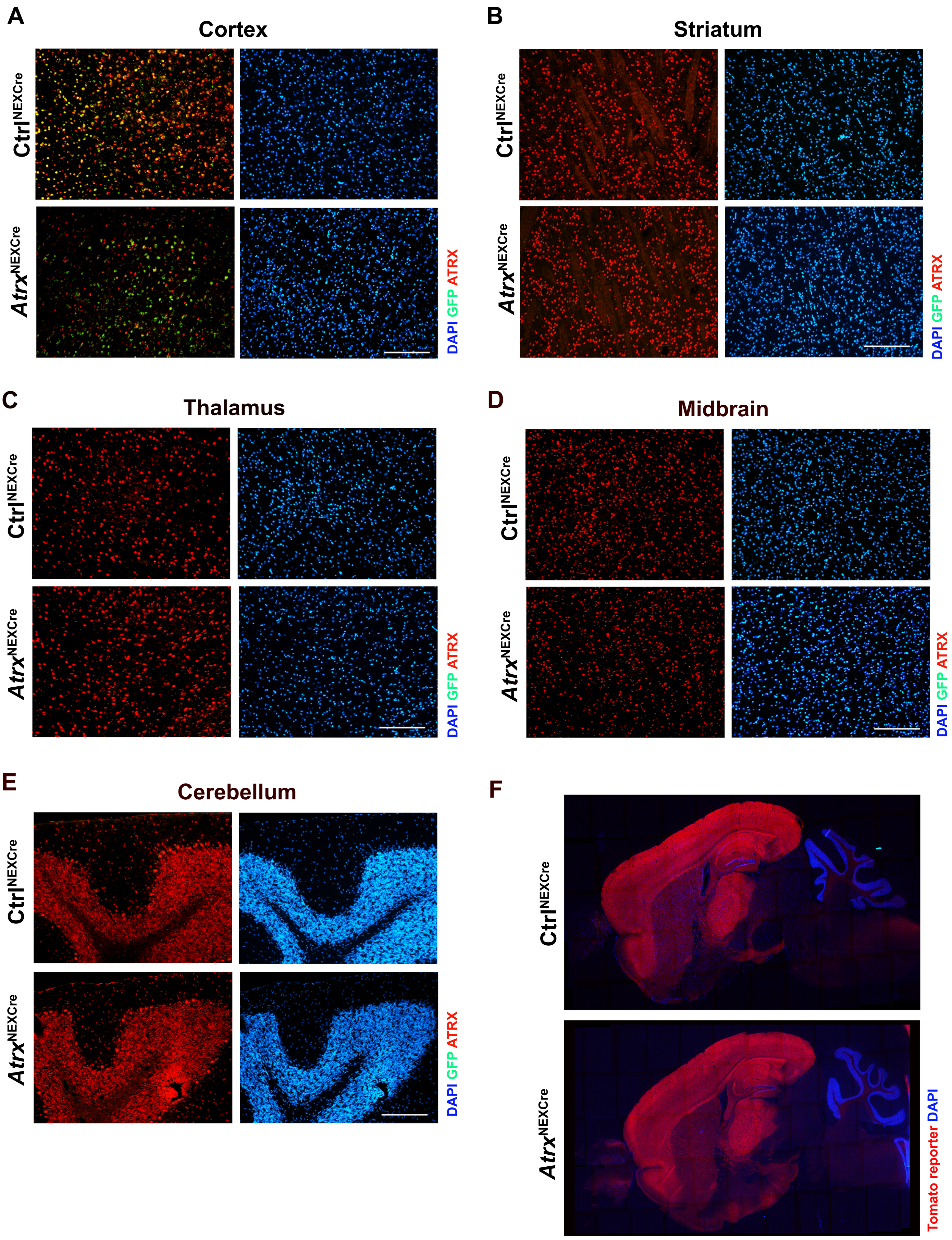

Supplemental Figure 4: Deletion of ATRX and Cre expression is specific to the forebrain. A) Cre-dependent expression of SUN1-GFP (green) is observed in nuclei of the cortex correlating with loss of ATRX (red) expression. **B)** The striatum, **C)** thalamus, **D)** midbrain and **E)** cerebellum nuclei do not express SUN1-GFP correlating with no loss of ATRX in these brain regions. (Representative images n=3, scale bar= 200um). **F)** Cre-dependent expression of tomato reporter labelled neurons, showing similar expression patterns between Ctrl^{NEXCre} and Atrx^{NEXCre} (representative images n=3).
